# Privacy rights versus surveillance, challenges of sex education in the digital age: an exploratory qualitative study in Indonesia

**DOI:** 10.1186/s12889-026-26281-z

**Published:** 2026-02-06

**Authors:** Nurmukaromatis Saleha, Restuning Widiasih, Iqbal Pramukti, Meita Dhamayanti, Titin Aprilatutini

**Affiliations:** 1 Doctoral Study Program, Faculty of Medicine, Padjadjaran University, Bandung, West Java Indonesia; 2 Nursing Department, Faculty of Mathematics and Natural Sciences, University of Bengkulu, Jl. Indragiri, Padang Harapan, Gading Cempaka, Bengkulu City, 38225 Indonesia; 3 Department of Nursing, Faculty of Nursing, Padjadjaran University, Bandung, West Java Indonesia; 4 Department of Child Health, Faculty of Medicine, Padjadjaran University, Jalan Raya Bandung-Sumedang KM. 21, Jatinangor, Sumedang, West Java 45363 Indonesia

**Keywords:** Curriculum, Electronics, Health promotion, Sex education, Students

## Abstract

**Background:**

Sexual health education has been globally recognized as one of the preventive factors in sexual violence prevention. Online child sexual exploitation and abuse (OCSEA) has emerged as a form of sexual violence against children facilitated by electronic media. This study aims to explore the experiences of parents, teachers, and public health center staff in providing sex education as a means of preventing OCSEA.

**Methods:**

An interpretative qualitative exploratory study was conducted on 32 participants selected by purposive sampling (10 parents of students, 16 school officials, 5 public health center officials, and 1 gender and family academics). Semi-structured interviews were conducted with 15 participants, and three focus group discussions were held with 17 participants from April to July 2025 in Bengkulu City, Indonesia. The interview guide was adapted from the health promotion model and school health promotion, and analyzed using thematic analysis.

**Results:**

Four themes with thirteen sub-themes were identified: 1) parenting dilemmas, 2) OCSEA as an iceberg phenomenon, 3) Preventive measures required, and 4) the importance of government and community support.

**Conclusion:**

Protecting children from OCSEA requires the implementation of comprehensive sexuality education integrated into the school curriculum in Indonesia. This study can be used as material for further studies related to OCSEA prevention interventions.

**Trial registration:**

Not applicable.

## Background

Digitalization, marked by increased internet usage, offers many benefits for children's growth and development, but it also poses potential dangers [1, 2]. One of these dangers is online child sexual exploitation and abuse (OCSEA) [3]. The United Nations (UN) has designated OCSEA as a global issue. The Sustainable Development Goals stipulate that children must be protected from all forms of violence, including OCSEA. OCSEA also poses a challenge to realizing children's rights, which were established 30 years ago [4]. OCSEA is a phenomenon related to internet use that encompasses all forms of sexual abuse against individuals under the age of 18 as victims, regardless of whether the victims are aware or unaware that such acts are abnormal and unacceptable [5]. These acts are typically perpetrated by adults or peers who exploit their power over the victim through deception and manipulation.

The internet has become an integral part of education due to its beneficial impact on improving the quality of education [6, 7]. Development theory suggests that the Internet, as part of technology, is a technological subsystem (microsystem) that can influence children's behavior [8]. Children have the right to a quality education and to be free from all forms of violence. Schools are places where children can carry out their developmental tasks. They spend nearly 65% of their time there [9–11]. Creating a child-friendly school environment (physical and online) can protect children from the risk of violence. As adults responsible for childcare, teachers and parents must collaborate to safeguard children from the dangers of OCSEA [12, 13]. One way to protect children from sexual abuse is to provide them with sex education [14, 15]. Although the internet can be a medium for informal sex education (e.g., TikTok, Facebook, Instagram), it also provides opportunities for various forms of online violence, such as cyberbullying and sexual violence. Informal sexual education is often not accurate or representative, lacking in portrayals of safe sex, because not all accounts have official and scientific sources [16]. This demonstrates that the roles of parents and teachers cannot be fully supplanted by the internet in the realm of sex education.

Previous studies have demonstrated the positive impact of parental and teacher involvement in sex education on preventing sexual violence [17–19]. However, studies investigating the role of both in protecting children from electronic-based sexual violence are still limited, especially in Bengkulu City, Indonesia. According to a survey by the Indonesian Internet Service Users Association, children and adolescents comprise the largest group of internet users [20]. In 2024, the Indonesian National Commission on Violence Against Women reported 1,687 cases of electronic-based sexual violence [21]. Previous health research has primarily focused on treating OCSEA-related trauma, with limited attention given to sexual education as a preventive measure.

In Bengkulu City, the Women and Children Protection Office includes reports of electronic-based sexual violence in its general sexual violence data, so there is no definitive data available. This data shows a discrepancy when compared to the results of a survey of 103 students conducted by the non-governmental organization Center for Women and Children's Education (PUPA) in 2023. The survey shows that 34% of students have experienced online gender-based violence [22]. Meanwhile, data from the protection agency indicates that there were 14 cases of sexual violence against children aged 13–17 years, without specifying the type of online or contact violence. Online gender-based violence of a sexual nature increases the risk of OCSEA. OCSEA can impact mental health [23]. Parents and teachers are at the forefront of children's education, while health workers at community health centers are at the forefront of health services [24, 25]. Therefore, health professionals need to address this issue through preventive measures. This study examines the experiences of parents, teachers, and health workers at community health centers in providing sex education as a means of preventing OCSEA.

## Methods

### Design

We conducted an exploratory and interpretive qualitative study to understand the threat of OCSEA from the perspectives of parents, teachers, and primary health care providers using a pragmatic approach [26, 27]. This exploratory and interpretive approach was adopted because the topic of sexual violence and sexual education, created within a sociocultural context, is an experience that tends to be hidden due to its association with social stigma and power relations, which can trigger feelings of fear, guilt, shame, ambivalence, and trauma. This is difficult to study in depth using positivist methods. An interpretive approach provides an opportunity for marginalized groups to express their views.

This study also adhered to the Declaration of Helsinki. Each participant first received a brief explanation of the purpose, benefits, and procedures of the study, as well as their right to participate without coercion and to withdraw at any time. Participant consent was recorded in a signed consent form. With this in mind, we took the following steps: 1) We anonymized participant accounts using separate codes for interviews (I) and focus group discussions (FGDs); 2) We removed all direct and indirect identifying information; 3) We modified all quotations that indicated individual, institutional, and location identification while maintaining the substantive meaning of the data. To prevent retraumatization, the researchers were sensitive to participants' distress, allowed them to refuse to answer questions, used non-blaming language, and allowed them to terminate the interview session. The researchers also provided information about relevant psychosocial support services and reporting mechanisms if discussions provoked emotional distress.

After obtaining ethical approval from the health research ethics committee of Padjadjaran University (registration number 2502050157; Number: 271/UN6.KEP/EC/2025), we proceeded to obtain permission from the Bengkulu provincial licensing office and then the Bengkulu provincial education office. We took permission from the education office to the principal of the school where the research would be conducted. The schools were selected based on the consideration that all three schools had excellent accreditation rankings and used the internet in their learning systems, Additionally, cases of sexual violence have been found in that location. The authors followed the consolidated criteria for reporting qualitative research studies (COREQ) checklist in presenting the results of this study [28].

### Participants

Teachers, parents, and health workers, and head of the Center for Gender and Family Studies at Bengkulu University were sampled using purposive sampling. We obtained teacher participants from the principal or vice principal (as the general person in charge at the school), who then assigned teachers, counselor, and school health workers. The teachers involved in this study taught subjects related to sexuality, such as biology, physical education, religion and morals. Parent participants were selected based on recommendations from teachers as key informants, with the criteria of being easy to communicate with and living with their children. The health workers who participated in this study were health workers at community health centers in the three school areas. These health workers were responsible for adolescent health and school health programs. Before visiting the health centers, we first obtained permission from the Bengkulu City Health Office.

The head of the Center for Gender and Family Studies at Bengkulu University as an expert participant. She was an expert participant from the University of Bengkulu with experience handling cases of sexual violence in schools.Expert participants are needed to reduce interpretive bias and increase the credibility of the research through theoretical triangulation [29]. Unlike the other participant groups, the head of the Center for Gender and Family Studies had a strong conceptual foundation. Additionally, she was relatively free from institutional pressures because he was not directly involved in schools or health centers. Table 1 shows an overview of the demographic data of the participants.

**Table 1:** Participant demographic data

Variabel	F	%
Parents (*n* = 10)		
Age		
35–50 y.o	9	90
> 50 y.o	1	10
Relationship with students		
Biological mothers	9	90
Grandmother	1	10
Education		
Senior High School	6	60
University	4	40
Job		
Housewife	7	70
Working women	3	30
School Authorities (*n* = 16)		
Age		
25–<35 (y.o)	3	18.75
35–50 (y.o)	8	50
> 50 y.o	5	31.25
Gender		
Woman	14	87.50
Man	2	12.50
Position		
Teacher with subjects (3 biology, 1 religion, 2 moral education)	6	37.50
Counselor	4	25
School health workers	6	37.50
Length of employment		
< 5 years	1	6.25
≥ 5 year	15	93.75
Last education:		
Bachelor's degree	2	12.5
Master's degree	14	87.5
Employment status		
Government employees	10	62.5
Temporary employees	6	37,5
The community health center (*n* = 5)		
Age		
35–50 (y.o)	4	80
> 50 y.o	1	20
Gender		
Woman	4	80
Man	1	20
Position		
Head of the community health center	2	40
School health program manager	1	20
Program manager youth health	2	40
Long tenure		
< 5 years	4	80
≥ 5 year	1	20
Head of the Center for Gender and Family Studies, University of Bengkulu (*n* = 1)		
Age	63 y.o	
Long tenure	5 years	

### Study setting

This study was conducted from April to July in Bengkulu City. Bengkulu City is the capital of Bengkulu Province. Although it is the capital, it has striking differences compared to the capitals of large provinces in Indonesia, such as Jakarta, Bandung, Surabaya, and Palembang. The difference in the field of education can be seen in the smaller number of schools compared to those cities, as well as in the scale of operations and facilities, which are still relatively lacking compared to large cities [30]. The level of digitization of schools in Bengkulu City is also still limited, for example, in the availability of digital devices and boards, and internet connectivity, which varies from good to limited. While learning platforms have been adopted, their penetration and intensity of use are not as high as in major cities due to limitations in infrastructure and human resources. In reality, these limitations do not shield children in Bengkulu City from the threat of OCSEA.

### Data collection

Data collection was conducted directly by the principal investigator from April 11 to July 8, 2025. The researcher and participants use Indonesian, and there are no language barriers. The principal investigator, a maternity nurse, had prior experience conducting qualitative research. The research team consisted of four people: three nurses (one maternity nurse, one community nurse, and one nurse with a master's degree in education) and one pediatrician. They all had professional experience in adolescent health promotion, which sparked their interest in sexual education issues. These backgrounds allowed the team to interpret data with an in-depth contextual understanding. Prior to data collection, the researcher assumed that schools had the potential to prevent and address OCSEA. The researchers have no personal or professional relationship with the participants.

We conducted focus group discussions (FGDs) with two groups: schools and community health centers. Within these groups, we examined the synergy between programs implemented to promote health, particularly those aimed at preventing sexual violence, in both online and offline spaces. The data explored was public data, not private. To the heads of community health centers, we gave them the option of participating in a focus group discussion or conducting face-to-face interviews, considering their busy schedules. In the end, we met with the heads of community health centers face-to-face. The FGD took place in a closed room to prevent unauthorised persons from entering. We visited each parent for a face-to-face interview. This was done because the requested data was related to their children's privacy. We hoped this method would make parents feel more comfortable revealing matters related to their children's upbringing and personal data. Interviews were conducted with the head of gender and family studies. For this study, the researcher conducted a pilot Focus Group Discussion first. The results of the pilot were not used as study findings.

Data saturation was achieved during the focus group discussion (FGD) when participants' responses revealed no new information and conveyed the same meaning. Participants agreed on the meaning. In interviews, saturation was achieved through data redundancy. There was no new information because any new data only served to strengthen existing themes. There were three FGD sessions, each attended by six, three, and four people, respectively (see Table 2). Participants who were unable to attend the FGDs were interviewed directly by researchers. All FGDs and interviews were conducted in a conducive room and lasted between 30–60 min. During the interview process, recordings were made, and photos and videos were taken.

**Table 2:** Data collection method

Method	Participant	Number
FGD 1	• counseling teacher • school health unit staff • public health center staff	1 2 3
FGD 2	• counseling teacher • school health unit staff	2 1
FGD 3	• school health unit staff • public health center staff • Teacher	2 1 1
Interview	• school health unit staff • head of the public health center • Teacher • Parents • gender and family academics	1 2 5 10 1

The research questions were developed from the school health promotion model and can be seen in the following Table 3.

**Table 3:** Interview guide for parent, teacher, and Public health center officer interview

Interviewee	Subject	Content/Possible questions
Parents	- Knowledge, perceptions, and attitudes about OCSEA	• What is your opinion on children's use of the internet and social media? • What do you know about online sexual violence against children? • What is your stance on the phenomenon of sexual violence in the online world?
	- Sex education	• How do you provide sexual health education to your children? • What are your thoughts on sex education as a means of protecting your children from the dangers of sexual violence in the online world?
	- Coordination with the school	• How do parents communicate with teachers at school? Have they ever discussed the prevention of online sexual violence? • Do you have any suggestions for schools regarding efforts to prevent online sexual violence?
Teachers	- Knowledge, perceptions and attitudes	• What is your opinion on the use of the internet for learning?
	- Experience	• Tell us about your experiences dealing with students who have problems in the online world, especially sexual violence
	- Preventive measures	• How do you prevent electronic-based sexual violence?
Public health center officer	- Knowledge about OCSEA	- Tell us about your experiences working with child sexual abuse
	- The Role and Efforts of Public Health Centers in Prevention	- Tell us what you know about online child sexual exploitation and abuse

### Data analysis and trustworthiness

Analysis is carried out after each data collection process is complete. The results of the interviews were transcribed verbatim and analyzed using six phases of thematic analysis iteratively [31]. Since this OCSEA topic had only a minimal conceptual framework and required further exploration, coding was carried out inductively, with themes developed directly from the data. This analysis was conducted manually with the help of Excel. Each group of participants was analyzed separately. The principal investigator familiarized herself with the data and made analytical notes, which were then used to create codes. Codes with similar essential meanings were combined as initial sub-themes that reflected the relevant literature. These codes were discussed with the author team to reach consensus, then categorized, sub-themed, and developed into themes. The themes developed from each participant group were compared to identify similarities, differences, and unique characteristics. They were then refined by creating a theme map and naming the themes so that their meanings were consistent with the research questions and could then be compiled into a report (see Table 3).

Rigorous processes are carried out to ensure the validity and reliability of the study [32, 33]. To avoid bias, during data collection, the principal researcher conducted bracketing and reflexive Journaling so that natural and objective data were obtained. The principal investigator always collaborates with other research teams that have expertise in methodology and substance (peer debriefing). The resulting theme map is sent to each research team for joint discussion (researcher triangulation). Triangulation of sources can be observed when counselors state that students who are exposed to sensual content without consent are not victims, but perpetrators of pornography. This is confirmed by the academic, who emphasizes the importance of school counselors receiving training in dealing with victims of violence with empathy. An example of method triangulation can be seen in the theme of parenting dilemmas. This theme emerged from interviews with parents and focus group discussions (FGDs), particularly in the form of statements from counsellors. This study draws on a range of theories, including health promotion, children's rights and social ecology.

## Results

Four themes and thirteen sub-themes were generated from the thematic analysis process (Table 3). The four themes are: 1) parenting dilemmas; 2) OCSEA as an iceberg phenomenon; 3) government and community support; and 4) the need for prevention efforts, as shown in Table 4.

**Table 4:** Study themes and subthemes

Themes	Group	Subthemes
1. Parenting dilemmas	• Parents • Teachers	1.1 Benefits versus potential threats of the internet 1.2 Fulfilling children's digital rights versus protecting children from online threats 1.3 Privacy rights versus surveillance 1.4 Freedom of expression versus pornography
2. OCSEA as an iceberg phenomenon	• Parents • Teachers	2.1 Stigma and victim blaming 2.2 Crime without a trace
3. Preventive measures required	• Parents • Teachers • Public Health Center staff • Gender and family academics	3.1 Training for school staff and teachers on the prevention and handling of sexual violence 3.2 School curriculum that integrates comprehensive sexuality education 3.3 Digital literacy and parenting training for parents and teachers
4. The importance of government and community support	• Parents • Teachers • Public Health Center staff • Gender and family academics	4.1 Funding constraints 4.2 Social media control 4.3 Coordination with relevant government agencies and non-governmental organizations 4.4 The role of public health center

### Parenting dilemmas

Parents, teachers, counselors, and school health unit staff agreed that we now live in an era where the internet is a necessity that offers many benefits. These benefits include access to various types of information, such as educational and health information, communication, socializing, entertainment, and an outlet for self-expression and participation. However, they also acknowledged the internet's negative impacts, including health risks, exposure to inappropriate content, and online sexual exploitation. This situation creates a dilemma for parents and schools, especially in their supervisory roles. This dilemma is exacerbated by the digital literacy gap between generations. Their statements demonstrate the internet's dual role as a social space for children, creating digital ambivalence. This situation requires ongoing negotiation so that children can weigh the internet's benefits and risks wisely.

#### Benefits versus potential threats of the internet

Participants stated that the internet aids in the learning and socialization processes of children. However, despite these benefits, the internet also poses various risks that can negatively impact physical and mental health. Therefore, open communication with children is essential.

“Actually, the internet has its good side and its bad side, depending on how you look at it. Maybe parents should explain to their children that cell phones are for learning. As far as I know, they are mostly used for WhatsApp and Facebook, but the important thing is that they can be used for communication.” student's grandmother, 60 years old (I).

“It affects health and eyesight, but it's useful for learning.” student's mother, 48 years old (I).

Although social media can increase social connectivity, it also opens up opportunities for harmful content and various forms of violence, including online sexual exploitation.

“Sometimes send indecent photos in that group.” student's mother, 36 years old (I).

“They first met on Facebook. Then they were invited to chat, video call, and send vulgar photos.” counselor, female, 57 years old (I).

#### Fulfilling children's digital rights versus protecting children from online threats

The impact of the Covid-19 pandemic has created a digital environment as a new normal, including in the field of education. Education is a universal right for childreen, digital-based education poses new challenges for parents and teachers. Parents expressed the importance of monitoring their children's internet usage. The following are statements from participants.

“It's not a problem if they hold their cell phones nowadays, because everything is on their cell phones, like lessons, storing everything, and so on. So just check their cell phones often, ma'am.” student's mother, 44 years old (I)..

“Nowadays, children inevitably spend a lot of time on their cell phones. So, as parents, we have to be able to manage our children's screen time and study time. We also have to be there for them when they need us.” Student's mother, 44 years old (I)..

“If we bring our phones to school, we use them in every class, especially biology. I don't just rely on textbooks for every subject.” Teacher of biology, female, 44 years old (I).

"So, we have to use the cellphone, yes, to use the internet, sometimes the facilities at school may not be complete." Teacher of moral education, female, 57 years old (I).

#### Privacy rights versus surveillance

Parental and teacher supervision can be challenging because cell phones are considered a child's personal property. Children have a right to privacy that adults must respect. Another challenge is that adults have less digital literacy than children. Participants stated:

“It's a bit difficult. We can't check it every day, and they have privacy, so it's impossible for us to check everything.” Student's mother, 44 years old (I).

Children have reflected on how they internalize digital rights related to mobile phone use. They are aware of the legal framework that limits others' access to their phones, including their parents'. This awareness is evident when children lock their phones, demonstrating their ability to independently manage their personal information. Ultimately, children can exercise this right to avoid adult supervision.

“Because our children are sometimes smarter than us, Ma'am. For example, if we want to look at their cell phones, they lock them. That's privacy, Ma'am, privacy. They know the law that says we can't look at their cell phones. There are privacy laws. Our children are smarter than us in that regard.” Teacher of moral education, man, 57 years old (I).

#### Freedom of expression versus pornography

The transition to adolescence presents parents with a dilemma between trust, freedom, independence, and protection. Teenagers do have freedom of expression in public spaces, including social media, but this must be done with caution, especially when it comes to pornography.

“Well, once kids enter high school, they're more mature and understand the situation, right? They know what they have to do, that's all.” Student's mother, 44 years old (I).

“Uploading a video on TikTok with a somewhat sensual dance that tended to emphasize her chest, the video recently went viral, resulting in us being summoned by the principal.” school health unit staff, female, 36 years old (FGD).

### OCSEA as an iceberg phenomenon

Several participants disclosed cases of OCSEA that occurred but were not reported to the authorities. This was due to social, cultural, psychological, and structural barriers. In addition to these, technological and legal barriers also prevented the disclosure of OCSEA cases. This situation is described as the tip of the iceberg because the number of cases on the surface is small compared to the total number.

#### Stigma and victim blaming

As a result of social stigma, feelings of shame and blame arise. Victims are often considered negligent and immoral, which obscures their status. The legal and ethical responsibility shifts from the perpetrator to the victim.

“The photos are everywhere. Her friends know because she was photographed naked. We also saw it at that time, in photos from head to toe.” Counselor, female, 57 years old (I).

“If we go back to the beginning, he wasn't actually a victim, ma'am. In fact, if she didn't like it, he should have just thrown it away.” counselor, female, 45 years old (FGD).

("thrown it away" is meant the sexual content; the statement suggests that the behavior was done out of love and consent, thus giving rise to the assumption of victim status is inappropriate).

The above statement suggests the counselor does not fully understand that child sexual abuse begins with the grooming stage. The following statement from the head of the Center for Gender and Family Studies at the University of Bengkulu reinforces this idea:

“I am a little concerned, pardon, that when counselors delve into cases of students who have experienced violence, there are things that actually reopen old wounds, both from the questions asked and perhaps from the attitudes shown, so that in the end, the child becomes even more ashamed, meaning they lose their self-confidence.” gender and family academics, 63 years old (I).

#### Crime without a trace

OCSEA cases are difficult to report due to the limited or nonexistent physical evidence, the ease with which it can be deleted or manipulated, and the difficulty of verifying it. These cases occur in the private digital sphere. The following participant quote demonstrates this:

“In disguise, impossible to catch…” Counselor, female, 57 years old (I).

“So, these perpetrators are not only school children, but also those outside the school environment.” counselor, female, 45 years old (FGD).

### Preventive measures required

OCSEA prevention efforts are recognized as a shared responsibility, not just the responsibility of individuals and families. As a second home for children, schools are considered a key factor in this effort.

#### Training for school staff and teachers on the prevention and handling of sexual violence

Teachers and staff are the next frontline school staff interacting with children at school, so they need to be equipped with the skills to recognize, prevent, and respond appropriately to sexual violence. Schools often focus solely on academic discipline, neglecting to recognize the early signs of sexual violence, both offline and online. Here are some statements from participants:

“Later, I will also propose in the meeting that we invite external speakers such as those from the Korin Center (training institute) again, because this is related to adolescent reproductive health.” Religion teacher, man, 53 years old (I).

“The role of counselor teacher should be preventive. They can approach students, meaning they can approach them to socialize them, to tell them to be careful, that they are now at a certain age, so the approach is more humanistic.” gender and family academics, 63 years old (I).

#### School curriculum that integrates comprehensive sexuality education (CSE)

In sexual violence prevention programs, the curriculum serves not only as a learning framework but also as a tool for child protection policies. Therefore, it is crucial to integrate comprehensive sexuality education (CSE) into the curriculum.

“In learning, we may not focus too much on prevention. We just relate it to the learning material, for example biology, or Pancasila, which is more about divine values. How to maintain self-respect, protect ourselves from falling into inappropriate content.” Teacher of moral education, man, 53 years old (I).

“In textbooks and reproductions, there are no precautions or anything like that. This is just for ourselves, because we are teachers, so how should we do it? So, before explaining the material, we must inform the children first.” Biology teacher, female, 44 years old (I).

“So, in my opinion, it needs to be included in the curriculum. How should this be handled so that prevention starts from elementary school, junior high school, and high school?” counselor, female, 45 years old (FGD).

#### Digital literacy and parenting training for parents and teachers

Parents and teachers must work together to prevent OCSEA. They must be able to act as digital advocates for their children. Limited technical skills can weaken supervision capabilities. Therefore, digital literacy must be strengthened, including an understanding of online risks, digital ethics, children's privacy rights, and non-repressive supervision strategies.

“There is still communication with the parents, but sometimes they don't respond to come. Sometimes they give the excuse that they are busy, which sometimes makes communication difficult.” Teacher of moral education, female, 57 years old (I).

“We have limitations. I don't understand, but kids nowadays understand, that's just how kids are. I'm the type of person who doesn't want to bother with cell phones. It's like if something changes, I panic. So it's the kids who often play with cell phones.” Student's mother, 36 years old (I).

### The importance of government and community support

OCSEA prevention efforts at the individual, family, and school levels require structural support from the government and community involvement. The synergy of all these components is a crucial factor in creating sustainable child protection.

#### Funding constraints

The main obstacle to preventing sexual violence and OCSEA is the lack of funding allocated to the programme.

“Especially since this comes from guidance counseling, religious teachers, Pancasila teachers like me, we must take the initiative, but that is one of the obstacles, in my opinion, namely finances. But I will try, perhaps in the form of new ideas, just plans, hahah... I don't know how it will turn out.” Religion teacher, man, 53 years old (I).

#### Social media control

The digital space is both cross-border and algorithm-driven, which makes it difficult to control. Controlling social media requires more than just user awareness — government intervention to oversee digital platforms is also necessary.

“The Ministry of Communication and Information Technology needs to know how people are, and which accounts are fake, right?” Counselor, female, 57 years old (I).

“With the Ministry of Communication and Information Technology, we hope that they can block inappropriate videos or images and stickers from reaching children's Android devices, and that there will also be education from the Ministry.” counselor, female, 44 years old (FGD).

Regulations restricting cell phone use in schools have become a subject of internal debate within schools. According to participants, addressing this issue requires policies to be made by both the central and local governments.

“In my opinion, ma'am, cooperation from the education sector is necessary. For example, the central government could also create a program on how to use social media wisely. Perhaps it should be included in the curriculum, ma'am. Because now it is a problem that all schools may face.” Counselor, female, 45 years old (FGD).

“We have that governor. Yes, that's possible. We teachers are having a hard time.” Counselor, female, 57 years old (I).

#### Coordination with relevant government agencies and non-governmental organizations

Sexual violence is a complex issue that requires cross-sectoral intervention. However, poor governance has resulted in programs not only overlapping but also interfering with each other, ultimately leading to failure.

“That's from the health department, we used to be active.” Biology teacher, female, 54 years old (I).

“I think that the National Population and Family Planning Agency or, for example, the Education Office should also be more proactive in educating these teenagers. Starting from the time they reach puberty. From junior high school and high school, it is very important, in my opinion.” “Corin Center, in the context of character education.” Teacher of moral education, man, 53 years old (I).

#### The role of public health center

In carrying out its duties, the Community Health Center does not have substantive autonomy but rather acts as a technocratic and procedural implementer:

“So, we made a plan to provide educational services, such as counseling. Then, we will check it according to technical guidelines.”

“These community health centers must comply with technical guidelines issued by the central government and the health department.” head of the public health center, woman, 36 years old (I).

Transformation in primary services by integrating services into clusters according to age groups. Reproductive health is in cluster 2. Community health centers have a regular agenda for schools. They visit with teams consisting of nutritionists, dental health professionals, mental health professionals, and adolescent reproductive health professionals.

“Well, now there is Cluster 2, so there is no longer a polyclinic. It covered children and teenagers. PKPR (Youth Care Health Services) are still running. We still carry out activities at school, such as counselling and distributing iron tablets, for example.” head of the public health center, man, 39 years old (I).

The inability of schools to recognize the early signs of sexual violence has led to communication barriers with community health centers. These barriers are also caused by the desire to maintain the school's image. During outreach and routine screening activities, health workers were able to identify these cases. Weak coordination between schools and community health centers resulted in late identification of sexual health issues, which ultimately developed into legal cases.

“Sometimes, schools may feel embarrassed, while we at the health center are called in. We were called in because at that time there was a case, a murder case. What year was that? The murder of a high school student by her boyfriend, who was burned. I was in charge of the PKPR program, and because the case involved the community health center, I was called in by the health office.”

“During counseling, there was one case, but several years ago, he should have been in junior high school, but he was still in elementary school. So I was confused as to why a 14-year-old child was still in elementary school. What was going on? Finally, I followed up on this case, and he ended up telling me about the sexual abuse committed by his stepfather.” Midwife, woman, health center staff in charge of adolescent health program, 56 years old (FGD).

The health center expects the active role of health workers in schools to become an extension of the health center to carry out preventive measures. One of the proposed preventive measures is health education through social media.

“For additional programs, after providing education... it is best if they are carried out by the school, namely by the school's Health Unit.” Midwife, woman, health center staff in charge of adolescent health program, 33 years old (FGD).

“Social media is easily accessible to school students, allowing them to access education directly from health centers.” Dentist, woman, health center staff responsible for the school health program, 28 years old (FGD).

## Discussion

The results of this study could significantly contribute to public health, particularly with regard to adolescent sexual health, violence prevention and the determinants of digital health. This study reveals that preventing child sexual abuse is not solely the responsibility of the individual. While previous studies have primarily focused on strengthening individual digital literacy, this study’s findings highlights the lack of systemic preventive coordination between schools, parents, and primary health services. Overall, the findings of this study reveal that sexual education is still considered taboo, leaving children vulnerable to OCSEA. Although government regulations exist, they are rarely disseminated or implemented. Indonesia's existing protection system remains fragmented, resulting in inadequate OCSEA prevention and treatment programmes.

### Parenting dilemma

The results of the study show that participants are aware of the dual role of electronic media, which provides benefits on the one hand and various disadvantages on the other. Participants revealed that the use of gadgets is a personal matter and a child's right to privacy, which can be taken to the bedroom, making supervision difficult. Almost all parents consider that teens in high school are capable of controlling themselves, and parents want to show their trust in their children. There is still very limited empirical evidence regarding the childcare dilemma in Indonesia. An integrative parenting model is required to balance protection from digital risks with respect for children's privacy, participation, and autonomy. This model involves active mentoring and digital literacy. In this model, parents act as supervisors and companions, offering children a sense of security and trust in the digital space. The Child Rights-Based Digital Parenting Model implements the Convention on the Rights of the Child and General Comment No. 25 (2021). The model has three main elements: Restrictive Mediation, Active Mediation, and Collaborative/Participatory Mediation [34, 35].

Parents and teachers acknowledge their lack of digital skills. Some of them try to acquire these skills in order to protect their children, but some parents resign themselves and give their trust while still urging their children to be careful when using social media. Disparities in digital skills are related to brain plasticity. Adolescents have higher brain plasticity than other age groups [36, 37]. Previous studies are in line with these results, with parenting dilemmas arising from participants' recognition of the many benefits of the internet, the need for adolescent autonomy, and the diversity of family environments in providing support and supervision [38–41]. Parents are their children's first teachers, so a protective role in digital education is essential [42, 43].

Schools are cautious about checking devices due to the potential for privacy violations. A teacher's statement that students have knowledge of the legal framework for privacy rights is certainly a good thing. However, this must also be balanced with comprehensive legal literacy. Although we have the right to freedom of expression, this right is not absolute and is subject to prevailing norms [44]. Children need to understand that pornography is harmful and does not constitute freedom of expression. For preventive and protective purposes, cell phone inspections are permissible under special circumstances. These inspections are carried out with good communication and legal ethics [45, 46]. Therefore, school authorities need to establish regulations regarding cell phone use in schools, including cell phone raids. Regulations regarding cell phone use in schools have been proven to improve student learning outcomes [47, 48]. This is necessary as a negotiation tactic to deal with students' strategies of resistance to adult supervision.

### OCSEA as an iceberg phenomenon

Based on the results of the exploration, it was found that in reality there were more cases than the data reported by the relevant agencies. The low reporting rate does not indicate a low incidence of KSBE. Victims and their families tended not to report the cases. To avoid embarrassment and stigma, they chose to transfer schools, which of course disrupted the students' learning process. A lack of knowledge about the meaning of online grooming leads people to view victims as perpetrators of pornography. In addition, perpetrators who use false identities and leave no evidence make it impossible for victims to report the case for investigation. Perpetrators are usually located far away, either outside the city or abroad. However, some perpetrators are known to the victim, such as the victim's boyfriend or family member. Emotional and social relationships are also factors that prevent victims from reporting.

Previous studies have shown that reporting OCSEA cases as a form of contact sexual violence faces obstacles in the form of stigma, social pressure, powerlessness, and trauma, which make victims and their families reluctant to get involved with the law [49–51]. OCSEA itself can potentially escalate into cases of contact sexual violence [52–54]. Online sexual violence is not always perpetrated by strangers; many cases are reported by people who are known to the victim [55]. This OCSEA phenomenon can have major psychological, social, and relational impacts, causing long-term trauma [56].

### Preventive measures required

The research findings suggest that school communities lack the knowledge and skills required to recognise the early signs of sexual violence, respond empathetically to victims and understand the reporting procedures. The findings also suggest that comprehensive sex education has not been implemented effectively. Teachers do counsel students on self-protection from sexual violence during lessons. However, there is no specific material integrated into sex-related subjects that discusses self-protection from sexual violence. Meanwhile, students have been learning online for several years due to the pandemic. This has resulted in inadequate material absorption. Specific materials on reproductive and sexual health are provided to students participating in extracurricular activities at the Adolescent Health Information Centre, but not all students take part. The materials on preventing sexual violence within these activities are also inadequate.

Efforts to prevent sexual violence in schools include strengthening resources and structures, which complement each other [57, 58]. These resources cover the whole school community, including teachers, counselors, staff, students, and security officers. To support these efforts, a dedicated team must be established to prevent and address all forms of violence in schools, as mandated by the 2023 Minister of Education Regulation Number 46 [59]. Urgent training is needed for school personnel, with teachers and counselors being prioritized as they interact directly with students. teachers and counselors are expected to play a dual role as both academic educators and key figures in creating a safe and responsive school environment that supports victims' recovery. Teachers play a vital role in shaping students' character, communicating with parents, monitoring technology use for threats of violence, and collaborating with internal and external school parties [60–62]. Training materials include guidance on teacher-student relationship boundaries, professional ethics, and legal obligations, thereby minimizing the risk of sexual violence and victim-blaming practices at school. To ensure good cooperation and synergy between teachers and parents, parents can also be involved in digital literacy training [63–65]. Furthermore, encouraging student participation as peer counselors strengthens resources. However, limited funds prevent this program from running optimally. Several studies have demonstrated the effectiveness of peer counselors in influencing their peers [66–68]. Explanations from peers are generally easier for students to understand than those from parents or teachers.

Improving the curriculum and promoting digital literacy are key to preventing sexual violence. Comprehensive Sexuality Education (CSE) is recognised globally as a fundamental human right. It includes a curriculum-based approach that teaches all aspects of sex: cognitive, emotional, physical and social. Comprehensive sex education among adolescents is expected to optimally improve sexual and reproductive health, increase respect for diversity, foster resilience in the face of risk factors and assist with decision-making and control over their own sexual behaviour, in a manner that is proportionate to their age and development and based on their best interests. CSE refers to the International Technical Guidelines on Sexuality Education (ITGSE), which were prepared by UNESCO in the revised 2018 edition [14, 69]. This education can be provided as separate lessons or integrated into the existing curriculum, with continuous provision from year to year [70–73]. Several previous studies have demonstrated the positive impact of CSE on adolescents' knowledge and attitudes towards sexual behaviour, effectively controlling adolescent sexuality [74–79].

### The importance of government and community support

Research results show that government support is essential for establishing a comprehensive and equitable child protection system. The same applies to support from non-governmental organisations. The government plays a crucial role in policymaking, funding, digital regulation, cross-sector coordination and optimising primary healthcare services. If these roles are implemented effectively, efforts to prevent sexual violence against children will become proactive rather than reactive [58, 80]. Funding constraints will undoubtedly hinder personnel training, infrastructure development, programme evaluation, and the long-term effectiveness of preventive interventions. This highlights the importance of community-based investment and interventions, as well as government support, [81–83].

Government control of social media to protect children and adolescents is crucial, given their vulnerable status. Children and adolescents are not yet emotionally mature and cannot think complexly enough to filter content presented on digital platforms. The Ministry of Communication and Information Technology plays a strategic role as a regulator of digital systems and cyberspace governance [84–88]. The Ministry of Communication and Information Technology should strengthen primary prevention through education and digital literacy, particularly in schools, by implementing training programmes. To comprehensively manage cyber violence, the Ministry should coordinate with relevant institutions across sectors, such as the Ministry of Women's Empowerment and Child Protection (KPPPA), the Ministry of Education and Culture, the police, and the Ministry of Health.

In general, research shows that interventions against sexual violence tend to be reactive rather than proactive and sustainable. The Ministry of Health plays a crucial role in preventing and addressing sexual violence through early detection, psychosocial support, public health education, and intersectoral coordination, provided by community health centers (Puskesmas) as frontline health service providers. However, Puskesmas have limited time to interact with students. Furthermore, programmes implemented by community health centres must comply with central technical guidelines. Consequently, existing peer counsellor training programmes can no longer be implemented as they are not covered by these guidelines. This situation highlights the importance of empowering school communities, particularly school health workers. School health workers are expected to function as extensions of Puskesmas, ensuring the sustainability of Puskesmas programs in schools [89].

The results indicate that the role of health workers, including school nurses, focuses solely on curative care, while their preventive role is not optimal. School nurses can play a role as educators. They must be able to build trusting relationships with their students. They are expected to provide a safe space for adolescents to share sensitive digital experiences, thereby facilitating early disclosure of OCSEA risks. Along with empowering school health workers, it is also necessary to improve their well-being, especially considering that they are all contract employees. Several previous studies have demonstrated the importance of optimizing school health workers to support health promotion programs. This aims to create a healthy and conducive school environment for the learning process [90–92].

Nurses are one of the health workers commonly recruited in schools. A fundamental characteristic of nursing care is a focus on meeting basic human needs continuously. This implies that adolescent nurses must be competent in identifying the unique needs of adolescents according to their age and be culturally adaptive. Nurses must be able to challenge taboo views about sexuality so that adolescents feel comfortable talking about it. This intervention can be developed into an intervention model with the following components: 1) ongoing therapeutic relationships, 2) digital psychosocial risk screening, 3) counseling for adolescents and families, 4) child rights-based health education, and 5) cross-sector coordination based on community health centers. This study has limitations in its generalizability. While the findings are credible within the context of the schools studied, their transferability to schools with different social characteristics and digital policies needs to be carefully considered.

## Conclusion

This study reveals that OCSEA is a complex issue involving the interaction of family dynamics, social systems, and digital ecosystems. Parents and teachers face a dilemma between protecting children and respecting privacy. Stigma and limited knowledge of reporting mechanisms mean many cases go unreported. OCSEA prevention requires collaboration between parents, teachers, the government, and the community. This collaboration should include strengthening digital literacy and comprehensive sex education, creating safe and responsive school environments, and strengthening systems for detection and referral. Government support in the form of digital regulation, funding, and cross-sectoral coordination, as well as community involvement in establishing social norms that reject violence and support victims, is essential for the sustainability of prevention efforts.

## Recommendation

This study addresses pressing issues related to OCSEA, filling a significant research gap in Indonesia. Interventions in primary care, particularly at community health centres (Puskesmas), require sustainability. This can be achieved by empowering school health workers and addressing their financial well-being. We recommend an OCSEA prevention model. This model should incorporate risk screening, brief counselling on safe digital practices and clear referral pathways to address identified gaps in structured prevention responses. Furthermore, resources and structures must be strengthened to support prevention programmes. One way to achieve this is by integrating CSE into the curriculum. In terms of the limited funding allocated to prevention programmes, schools must optimise existing resources, for example by providing education through social media. As community health centres cannot work alone in prevention efforts, other relevant sectors, particularly the Ministry of Communication and Information Technology, must also be involved. Further research is needed to obtain effective, evidence-based interventions.

## Data Availability

The data (in Indonesian) used and/or analyzed during the current study are available from the corresponding author upon reasonable request.

## Abbreviations

CSE: Comprehensive sexuality education
ECPAT: End child prostitution, child pornography and trafficking of children for sexual purposes
ITGSE: International technical guidance on sexuality education
OCSEA: Online Child Sexual Exploitation and Abuse
